# Metabolomic signatures of high-intensity and sprint interval exercise/training in humans: a systematic review

**DOI:** 10.1007/s11306-025-02385-2

**Published:** 2026-02-09

**Authors:** Daniel Marques de Sá e Silva, Glykeria Avgerinou, Anatoli Petridou, Georgios Theodoridis, Vassilis Mougios, Helen Gika

**Affiliations:** 1 School of Chemistry, Aristotle University of Thessaloniki, Thessaloniki, 54124 Greece; 2 Biomic AUTh, Center for Interdisciplinary Research and Innovation (CIRI- AUTH), Balkan Center, B1.4, Thessaloniki, 57001 Greece; 3 School of Physical Education & Sport Science at Thessaloniki, Aristotle University of Thessaloniki, Thessaloniki, 57001 Greece; 4 School of Medicine, Aristotle University of Thessaloniki, Thessaloniki, 54124 Greece

**Keywords:** Exercise metabolism, HIIT/HIIE, Metabolomics, Pathway analysis, SIT/SIE

## Abstract

**Background:**

Exercise metabolomics investigates how physical activity alters the metabolome, with responses depending on exercise type, intensity, and duration. Intermittent high-intensity to supramaximal exercise produces unique metabolomic effects that remain inadequately addressed in the literature.

**Aim of review:**

This study aimed to (i) conduct a systematic review of publications on metabolomics, applied to high-intensity interval exercise or training (HIIE/HIIT) or sprint interval exercise or training (SIE/SIT) protocols in humans and (ii) provide an overview of the most characteristic metabolomic changes induced by these types of exercise.

**Key scientific concepts of review:**

A total of 20 studies met the inclusion criteria, with a variety of participants, biological samples, sampling procedures, and metabolomic analysis techniques. Pathway analysis revealed that the affected pathways were mostly related to carbohydrate, lipid, and amino acid metabolism. The tricarboxylic acid cycle and purine degradation were also considerably affected. Most metabolites were upregulated by HIIE/HIIT and SIE/SIT. Our analysis revealed strong and wide metabolomic changes with HIIE/HIIT or SIE/SIT, with substrate utilization for energy production emerging as a recurring theme. Such results suggest that the metabolic changes caused by exercise cannot be covered by a single analytical technology and underline the importance of reproducibility and the need for better control of modulating/confounding factors in future studies.

**Supplementary Information:**

The online version contains supplementary material available at 10.1007/s11306-025-02385-2.

## Introduction

In biological sciences, several fields of study enable us to understand the molecular dynamics of the constituents of life. Over the past twenty years, technological advances in mass spectrometry, nuclear magnetic resonance spectroscopy, and bioinformatics have led to the establishment of metabolomics as one such important field. Given the complexity and intricacy of biological systems, metabolomics can serve as a powerful tool for achieving a better understanding of the phenotypic changes caused by internal and external factors, such as diet, disease, drugs, and exercise (Idle & Gonzalez, 2007; Jaguri et al., 2023).

Physical activity, exercise, and sports participation are widely recognized as powerful modulators of human physiology and biochemistry. Changes at the molecular level are both short- and long-term. Elucidating these changes helps explain many of the established beneficial effects of exercise on the cardiovascular, respiratory, muscular, skeletal, endocrine, and other systems. This is why exercise metabolomics, that is, the study of the responses of the metabolome to exercise, can be a valuable tool for our in-depth understanding of the molecular mechanisms underlying the effects of exercise.

Like many other exercise-induced changes, metabolomic responses depend highly on the exercise protocol used (Jaguri et al., 2023; Sakaguchi et al., 2019; Schranner et al., 2020). Factors like exercise type, intensity, and duration exert profound effects on the differences in metabolite concentrations measured between pre- and post-exercise samples (Muscella et al., 2020; Schranner et al., 2020). An additional factor, exercise format, comes into play when exercise is intermittent, rather than continuous. In such cases, the intensity and duration of the exercise bouts, but also the intensity and duration of the rest intervals, may affect the metabolomic changes (Peake et al., 2014; Pechlivanis et al., 2010; Zafeiridis et al., 2016). However, few studies have directly compared protocols with different bout or rest characteristics; thus, the specific impact of these factors remains less well explored than the metabolomic changes associated with continuous exercise (Bogdanis et al., 2022a, b; Danaher et al., 2016; Darragh et al., 2023; Pechlivanis et al., 2015; Wang et al., 2023; Zafeiridis et al., 2016). Two exercise modalities characterized by intermittent exertion are high-intensity interval exercise/training (HIIE/HIIT) and sprint interval exercise/training (SIE/SIT) (Mougios, 2019). The former involves submaximal efforts eliciting at least 80% of the maximal heart rate (HRmax), while the latter involves all-out efforts or an intensity corresponding to at least 100% of the power corresponding to maximal oxygen uptake (V.O_2_max) (Mougios, 2019). Training involves multiple exercise sessions.

The rapidly growing number of studies on exercise metabolomics over the past 20 years (Lv et al., 2022) has brought several narrative and systematic reviews on the topic, mainly focusing on study design, analytical methodology, bibliometrics, and challenges faced by the field (Carrard et al., 2022; Daskalaki et al., 2014; Sakaguchi et al., 2019; Schranner et al., 2020). Of the systematic reviews that have focused on the findings of exercise metabolomic studies, Schranner et al. (2020) summarized studies on the effects of a single bout of endurance or resistance exercise published up to 2018, Sakaguchi et al. (2019) reviewed studies on the effects of various exercise types up to 2019, and Carrard et al. (2022) dealt with studies on cardiopulmonary exercise tests up to 2021. Finally, Daskalaki et al. (2014) conducted a non-systematic review of studies on the effects of various exercise types up to 2014. None of the reviews focused on changes in the metabolome after intermittent exercise of high to supramaximal intensity, which has been shown to elicit biochemical and physiological effects distinct from those of continuous exercise of the same duration and average intensity (Bogdanis et al., 2022; Bogdanis et al., 2022).

Therefore, the present work is a systematic review of publications on metabolomics applied to HIIE/HIIT and SIE/SIT. We review metabolomic results for the 2 modalities separately and combined by means of metabolic pathway analysis.

## Methods

### Study selection criteria

The inclusion criteria that were applied to the literature survey were the following:

- Studies on exercise interventions that applied metabolomic approaches to identify changes in metabolite concentrations as a response to exercise.
- Studies that used spectroscopic/spectrometric techniques, i.e., nuclear magnetic resonance spectroscopy (NMR) or mass spectrometry (MS) in blood, urine, or muscle-derived samples.
- Studies on healthy human subjects, including overweight and obese.
- Studies including sampling before and after HIIE/HIIT or SIE/SIT interventions.
- All articles published up to December 2024 in English.

### Search methodology

**Fig. 1:**
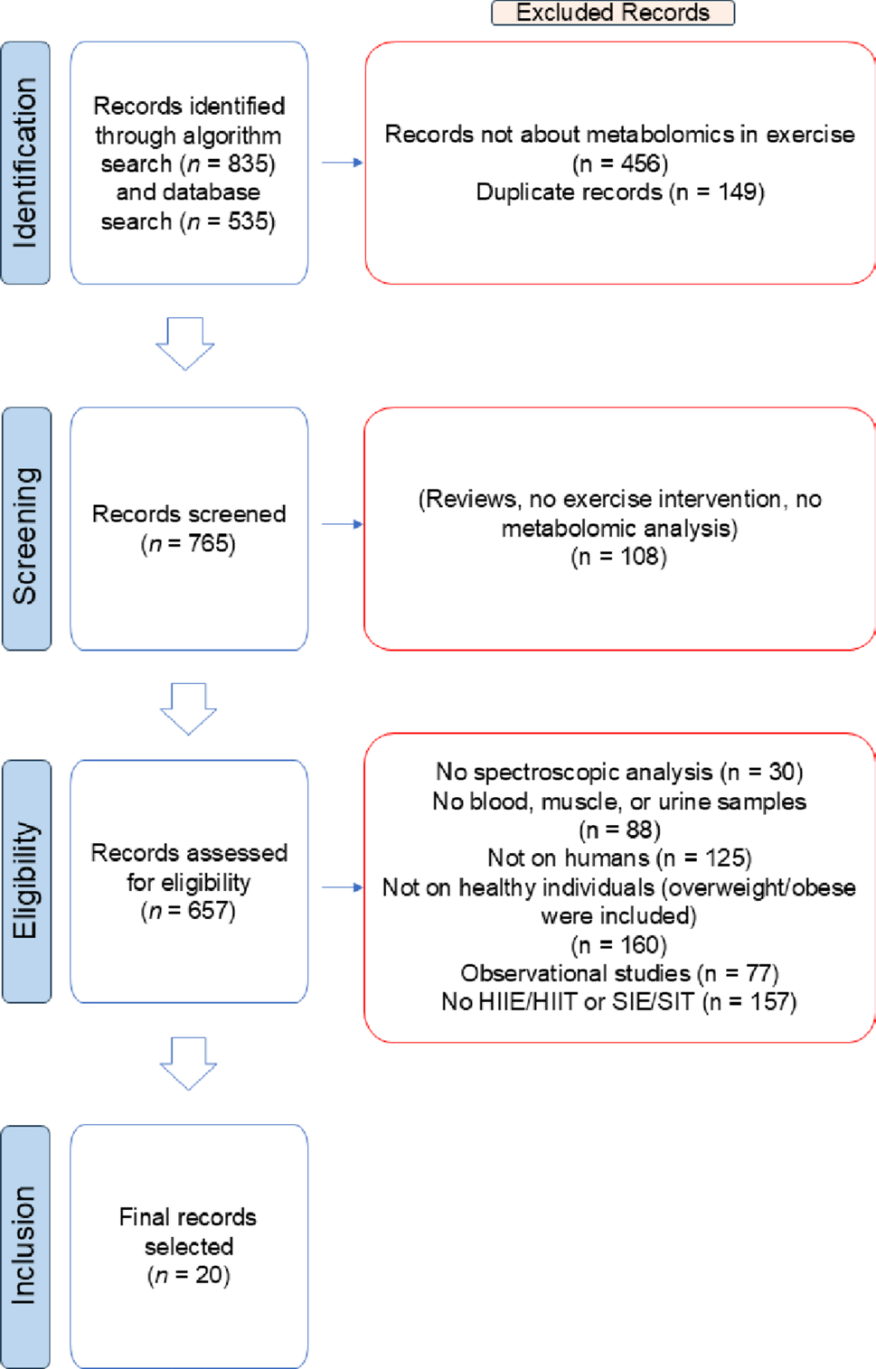
Flow diagram of study selection, showing identification, screening, eligibility, and final study inclusion

The detailed process of study selection is summarized in a PRISMA flow diagram (Fig. 1). A bibliographic search was conducted independently by the first two authors in two ways. The last search was performed in March 2025. First, a bespoke algorithm was used for literature mining, searching the PubMed database with the following command: (metabolic profiling OR metabolome OR global profiling OR lipidomic OR metabolic fingerprint OR metabolomic OR metabonomic OR lipid profiling OR metabolic phenotype OR metabolic phenotyping OR metabolic marker OR metabolite (bio)marker) AND (physical exercise OR training OR physical activity OR sports). This search retrieved 835 papers. Second, public databases (PubMed, Scopus, and Google Scholar) were searched using these keywords: metabolomics AND exercise OR training OR physical exercise. This search retrieved 535 papers. Overall, this step retrieved a sum of 1370 entries. The 2 authors independently conducted a preliminary check of the entries by reviewing each title and abstract to exclude any studies which did not address the subject of metabolomics in exercise. In addition, duplicate studies (i.e., studies found in both searches) were identified and only 1 entry was selected. This identification procedure resulted in 765 articles.

Subsequently, a screening was performed to remove 108 reviews and studies without an exercise intervention or metabolomic analysis. The remaining 657 articles were assessed for eligibility by checking if they matched the inclusion criteria. This resulted in 20 articles, which were included in the analysis. Significant differences reported below from these studies are at *p* < 0.05 or lower.

### Pathway analysis

To examine which metabolic pathways were most affected by HIIE/HIIT or SIE/SIT, pathway analysis was performed with MetaboAnalyst 6.0 (XiaLab, McGill University, Montreal, Canada). Plots were built by feeding the HMDB ID of the analytes whose concentrations had been reported to change with exercise or training into the software. The enrichment method for the over-representation analysis was hypergeometric test, with relative-betweenness centrality as the pathway topology measure. The Human KEGG pathway library (Version December 2024) was used as reference.

## Results

### Overview of the studies

The 20 selected studies were conducted between 2010 and 2024. Details of these studies are described in Tables 1 and 2. They included 328 human participants aged 12 to 70. Fourteen studies involved only males (*n* = 213, ranging from 7 to 34 per study, ln 2,4–7 in Tables 1 and 1–10 in Table 2), two involved solely females (*n* = 20; 11 and 9, respectively, ln 1 and 3 of Table 1), and four included both male (*n* = 45, ranging from 8 to 13 per study) and female participants (*n* = 40, ranging from 8 to 13 per study, ln 8–11 of Table 1). Eleven studies used HIIE/HIIT and nine used SIE/SIT.

A variety of biological samples were used in these studies. Specifically, solely plasma was used in seven studies (ln 1–4,8 in Tables 1, 4, 5 and 10 in Table 2), serum in five (ln 7 and 9 in Tables 1, 2, 8 and 9 in Table 2) and urine in five (ln 5 and 6 in Tables 1 and 1, 3 and 7 in Table 2). One study used capillary blood (ln 10 Table 1), one used muscle samples (ln 11 in Table 1), and another used serum and muscle samples (ln 6 in Table 2).

Various techniques were used in the analysis of these samples. Specifically, gas chromatography coupled to MS (GC-MS) was applied in two studies (ln 2 of Table 1 and ln 4–5 of Table 2), liquid chromatography coupled to MS (LC-MS) in eight (ln 6,8 and 9 in Tables 1 and 6–10 in Table 2), capillary electrophoresis coupled to MS (CE-MS) in two (ln 1 and 3 in Table 1), and NMR in four (ln 4 and 7 in Tables 1 and 1–2 in Table 2). Additionally, two studies employed a combination of LC-MS and NMR (ln 5 in Table 1 and ln 3 in Table 2), while two others used a combination of LC-MS and GC-MS (ln 10–11 in Table 1). Regarding the type of analysis performed, seven studies performed targeted analysis, ten performed untargeted analysis, and three employed both targeted and untargeted methodologies. Regarding the untargeted analysis, papers which explicitly point out the use of standards for annotations have been assigned a MetID level 1.

Dietary protocols varied noticeably across studies, ranging from strict control to free-living conditions. Some studies used standardized protocols to minimize the influence of diet on the results. Peake et al. (2014) asked participants to abstain from alcohol and caffeine, report dietary intakes through food diaries, and consume a standardized pre-exercise meal. Zafeiridis et al. (2016) used a controlled diet (55% carbohydrate, 30% fat, 15% protein) for two days before testing, with a standardized pre-test meal and restrictions on alcohol and caffeine. Likewise, Pechlivanis et al. (2010, 2013, 2015) implemented dietary plans with 50% carbohydrate, 35% fat, and 15% protein two days before testing and standardized pre-test meals. Danaher et al. (2016) repeated meals based on food records, with morning test performed in a fasted state. Dong et al. required participants to abstain from alcohol, coffee and supplements for 48 h before testing and provided a standardized breakfast on the morning of test day. Pataky et al. (2024) included three days of weighted meals (50% carbohydrate, 30% fat, 20% protein), followed by overnight fasting prior to testing.

Other studies preferred maintaining a habitual diet with varying levels of monitoring. Aird et al. (2021) restricted caffeine and alcohol before testing but otherwise instructed participants to maintain habitual intake and collected a 7-day weighted food diary. Cruz et al. (2022) had participants replicate their 24-hour dietary intake across sessions with a 2-hour pre-test fast. Jurado-Fasoli et al. (2022), Youssef et al. (2023), Zhou et al. (2022), Darragh et al. (2023), and Wang et al. (2023) also maintained habitual dietary intakes assessed by three-day food records or food diaries and recalls, with additional restrictions on alcohol and caffeine. Finally, several studies reported minimal or no dietary control (Karlsson et al., 2024; Kistner et al., 2019; Kuehnbaum et al., 2014, 2015b), while Zhao et al. (2020) provided no details on diet.

Below is a presentation of the highlights of each study, also presented in Tables 1 and 2.

**Table 1:** Studies on HIIE/HIIT metabolomics; design and main findings

	Authors	Participants	Intervention	Biological samples	Method	Analysis - metabolites	MetID identification level	Significant changes post- vs. pre-intervention
1	Kuehnbaum et al. (2014)	11 overweight/ obese sedentary women	HIIT: 6 weeks, 18 sessions of 10 1-min cycling bouts at 90% HRmax	Plasma at rest, pre- and 72 h post-training	CE-MS	Untargeted	2	Ornithine, proline betaine, trimethyllysine Cysteinylglycine-cysteine disulfide, glutathione-cysteine disulfide, cystine
2	Peake et al. (2014)	10 trained male cyclists and triathletes	HIIE: 10 4-min cycling bouts at 82% VO_2_max	Plasma pre- and immediately, 1 h, and 2 h post-exercise	GC-MS	Targeted (49 metabolites)	2	*Immediately post-exercise* Glucose, lactate, citrate, aconitate, succinate, malonate, 3-methyl-2-oxovalerate, 2-oxoisocaproate, NEFA (10:0, 12:0, 14:0, 14:1, 16:1, 17:1, 18:1), alanine, glutamate, tyrosine *During recovery (compared with pre-exercise)* ● Leucine, valine, isoleucine, methionine, alanine, proline ● NEFA (14:0, 16:1)
3	Kuehnbaum et al. (2015)	9 overweight/obese sedentary women	HIIT: 6 weeks, 18 sessions of 10 1-min cycling bouts at 90% HRmax	Plasma pre-, and 20- and 40 min post-exercise in the 1 st and 17th sessions	CE-MS	Untargeted	2	*Acute exercise effects* Acetycarnitine, hypoxanthine *Training effects* Carnitine glutathione-cysteine disulfide at rest hypoxanthine post-exercise
4	Zafeiridis et al. (2016)	9 young trained men	HIIE: ca. 5 3-min runs at 95% MAV or ca. 18 30-s runs at 110% MAV	Plasma pre- and 5 min post-exercise	NMR	Untargeted	2	Pyruvate, glucose, alanine, lactate, glycerol, citrate, succinate
5	Kistner et al. (2019)	10 trained men	HIIT: 10 days, 10 sessions of 8 cycling bouts at Pmax, each about 97 s	Urine at rest pre- and 1 and 4 days post-training	LC-MS, NMR	Targeted (28 metabolites by LC-MS) Untargeted	1 (targeted analysis, including betaine) and 2	*1 day post-training* Hypoxanthine *4 days post-training* Betaine, hypoxanthine, isoleucine
6	Zhao et al. (2020)	23 male soccer players	HIIE: Yo-yo test (repeated 2 × 20-m shuttle runs to exhaustion)	Urine pre-and 30 min and 18 h post-exercise	LC-MS	Untargeted	2	*30 min post-exercise* Arginine, proline, glutamate, citrulline, *N*-acetylornithine, meso-DAP, *N*-acetyl-DAP, γ-glutamylputrescine, 2-oxo-4-hydroxy-5-aminovalerate, 4-hydroxyglutamate semialdehyde Androstenedione, estrone
7	Cruz et al. (2022)	15 physically active men	HIIE: 10 1-min cycling bouts at 100% VO_2_max	Serum pre- and 10 and 60 min post-exercise	NMR	Untargeted	2	*10 min post-exercise* 2-hydroxyvalerate, 3-methyl-2-oxovalerate, *N*-acetylserotonin, oxypurinol, valerate Acetone, carnitine, lysine, thymine *60 min post-exercise* Hypoxanthine, oxypurinol 3-Methylxanthine, acetone, lysine, succinylacetone
8	Jurado-Fasoli et al. (2022)	17 middle-aged sedentary adults (8 men, 9 women)	HIIT: 12 weeks, 24 sessions of running and weight-bearing exercises, lasting 40–65 min	Plasma at rest, pre- and 72–96 h post- training	LC-MS	Targeted (50 oxylipins derived from ω6 PUFA, 28 oxylipins from ω3 PUFA, 10 endocannabinoids)	1	No difference in any metabolite from a non-training group.
9	Youssef et al. (2023)	26 obese sedentary older adults (13 men, 13 women)	HIIT: 12 weeks, 36 sessions of 10 30-s bouts at 80–85% HRmax on elliptical device	Serum at rest, pre- and post- training	LC-MS	Untargeted	1 (*n* > 200) and 2	2-Oxoglutarate, fumarate, 2-oxoisovalerate, stearoylcarnitine, 17:0 NEFA 3-Methylhistidine, aspartate, acetate, glycerate, 2-hydroxybutyrate, 2-oxovalerate, inosine, acetylcarnitine, d18:1/24:0 ceramide, DG (18:1/18:3, 20:4/18:2), isobutyrate, 18:2 NEFA, 16:0 PCae, 22:1 PCae
10	Karlsson et al. (2024)	23 trained cross-country skiers (13 men, 10 women)	HIIE: 5–6 graded 4-min roller skiing bouts, followed by a ~ 3-min time trial	Blood pre- and post-exercise	GC-MS, LC-MS	Targeted (24 metabolites), untargeted	1 (*n* = 26) and 2	Lactate, pyruvate, uridine, malate, succinate, uracil, glycerate, xanthosine, hypoxanthine, inosine, ribose 5-phosphate, xanthine, glucose 6-phosphate, fructose 6-phosphate, cyclic AMP, octanoylcarnitine, decanoylcarnitine, *cis*−4-decenoylcarnitine, lauroylcarnitine, 2-hydroxydodecanoylcarnitine, *trans*−2-dodecenoylcarnitine, myristoylcarnitine, 3,5-tetradecadienoylcarnitine, 9,12-hexadecadienoylcarnitine 3-phosphoglycerate, phosphoenolpyruvate, AMP, uridine 5’-monophosphate, deoxycholate
11	Pataky et al. (2024)	19 sedentary older adults (11 men, 8 women)	HIIT: 12 weeks, 36 sessions of 4 4-min cycling bouts at > 90% HRmax	Muscle at rest, pre- and 72 h post-training	GC-MS, LC-MS	Targeted (72 metabolites), untargeted	1 (targeted) and 2	Citrate, 2-oxoglutarate, 2-hydroxyglutarate, succinate, fumarate, malate, glutamate, phenylalanine, γ-aminobutyrate, aspartate, asparagine, methionine, serine, leucine, isoleucine, valine, octanoylcarnitine, lauroylcarnitine, myristoylcarnitine, palmitoylcarnitine, stearoylcarnitine, oleoylcarnitine, sphingosine, ceramides (d18:1/14:0, d18:1/16:0, d18:1/18:0, d18:1/22:0) Lysine, citrulline, sarcosine, 1-methylhistidine, carnitine, propionylcarnitine, isovalerylcarnitine

AMP, adenosine monophosphate; CE, capillary electrophoresis; DAP, diaminoheptanedioate; DG, diacylglycerol; GC, gas chromatography; HIIE, high-intensity interval exercise; HIIT, high-intensity interval training; HRmax, maximal heart rate; HRR, heart rate reserve; LC, liquid chromatography; MS, mass spectrometry; NEFA, non-esterified fatty acids; NMR, nuclear magnetic resonance spectroscopy; PCae, acyl-alkyl-phosphatidylcholine (the 16:0 and 22:1 notations refer to the alkyl moiety; the acyl moiety was not determined); Pmax, maximal power; PUFA, polyunsaturated fatty acids; VO_2_max, maximal oxygen consumption; , increase; , decrease

**Table 2:** Studies on SIE/SIT metabolomics; design and main findings

	Author	Participants	Intervention	Biological samples	Method	Analysis-metabolites	MetID identification level	Significant changes post- vs. pre-intervention
1	Pechlivanis et al. (2010)	12 young, moderately trained men	SIE: 3 sets of 2 80-m maximal runs with 10 s–1 min rest between twin runs	Urine, pre- and 35 min post-exercise	NMR	Untargeted	1 and 2	Lactate, 2-hydroxyisovalerate, 2-oxoisocaproate, 3-hydroxyisobutyrate, 3-methyl-2-oxovalerate, 2-oxoisovalerate, 2-hydroxybutyrate, alanine, pyruvate, 2-oxoglutarate, inosine, fumarate, hypoxanthine Citrate, trimethylamine *N*-oxide, taurine, glycine, allantoin, phenylalanine, hippurate, tryptophan, formate *Metabolites with higher change in runs with 10-s* vs. *1-min interval* Lactate, 2-hydroxybutyrate, 2-oxoisocaproate, 3-methyl-2-oxovalerate, 2-oxoisovalerate, alanine, pyruvate, citrate, 2-oxoglutarate, fumarate *Metabolites with higher change in runs with 1-min* vs. *10-s interval* Glycine, hypoxanthine
2	Pechlivanis et al. (2013)	14 young, moderately trained men	SIT: 8 weeks, 24 sessions of 2 (first 4 weeks) or 3 sets (last 4 weeks) of 2 80-m maximal runs	Serum, before and 30 min after a session of the first and the last weeks	NMR	Untargeted	1 and 2	*Acute exercise effects* Lactate, pyruvate, alanine Leucine, valine, isoleucine, arginine/lysine, glycoprotein acetyls *Training effects* Methylguanidine, citrate, glucose, valine, taurine, trimethylamine *N*-oxide, choline-containing compounds, histidine, 1-methylhistidine, 3-methylhistidine, acetoacetate/acetone Lactate, pyruvate, glycoprotein acetyls, lipids
3	Pechlivanis et al. (2015)	17 young, physically active men	SIE: 2 sprint sessions of 3 80-m maximal runs	Urine, pre- and 1, 1.5, and 2 h post-exercise	NMR, LC-MS	Untargeted	1 and 2	*1 h post-exercise* Lactate, 2-hydroxyisovalerate, 2-hydroxybutyrate, 2-oxoisocaproate, 3-methyl-2-oxovalerate, 3-hydroxyisobutyrate, 2-oxoisovalerate, 3-hydroxybutyrate, 2-hydroxyisobutyrate, alanine, pyruvate, fumarate, acetate, hypoxanthine, inosine Urate, valine, succinate, citrate, trimethylamine, trimethylamine *N*-oxide, tyrosine, formate, creatinine, glycine, 4-aminohippurate, hippurate *1.5 h post-exercise* Lactate, acetate, hypoxanthine, inosine, urate Valine, isoleucine, succinate, methylamine, trimethylamine *N*-oxide, tyrosine, formate, creatinine, glycine, 4-aminohippurate, hippurate *2 h post-exercise* Hypoxanthine, inosine Creatinine, glycine, 4-aminohippurate, hippurate
4	Danaher et al. (2016), 1 st protocol	7 physically active men	SIE: 30 20-s cycling bouts at 150% VO_2_peak	Plasma, pre-, during, immediately post-, and 1 h post- exercise	GC-MS	Untargeted	1	*During exercise* Lactate, malate, citrate *Immediately post-exercise* Lactate, malate, citrate, alanine *1 h post-exercise* Malate, citrate, xylose Sorbose, cholesterol
5	Danaher et al. (2016), 2nd protocol	7 physically active men	SIE: 30 10-s cycling bouts at 300% VO_2_peak	Plasma, pre-, during, immediately post-, and 1 h post- exercise	GC-MS	Untargeted	1	*During exercise* Lactate, malate, cholesterol Citrate *Immediately post-exercise* Lactate, malate, sorbose, fructose, cholesterol Asparagine, NEFA (16:0, 18:0), erythronate, xylitol, xylose *1 h post-exercise* Lactate, alanine Glutamate, asparagine, lysine, NEFA (12:0, 16:0, 18:0), erythronate, xylitol, xylose
6	Aird et al. (2021)	28 young, moderately trained men	SIT: 3 weeks, 9 sessions of 4–6 30-s maximal cycling bouts	Serum at rest, pre-, immediately and 1 h after the 1 st session; muscle tissue at rest and 3 h after the 1 st session; serum and muscle tissue 48–72 h post-training	LC-MS	Targeted (173 metabolites)	1	No statistical analysis of the effects of exercise or training (analysis only of the impact of protein supplementation)
7	Zhou et al. (2022)	12 physically active men	SIE: 3 30-s maximal cycling bouts	Urine, pre- and 50 min post-exercise	LC-MS	Targeted (18 metabolites)	1	No statistical analysis of the effects of exercise or training (analysis only of the impact of bicarbonate supplementation)
8	Darragh et al. (2023)	34 physically active men	SIT: 3 weeks, 9 sessions of 4–6 30-s maximal cycling bouts	Serum at rest, pre- and 48–72 h post-training	LC-MS	Targeted (168 metabolites)	1	NEFA (14:0, 15:0, 16:0, 16:1, 17:0, 18:0, 18:1, 18:2, 18:3ω3, 18:3ω6, 20:3)
9	Wang et al. (2023)	12 untrained male adolescents	SIT: 6 weeks, 18 sessions of 4–6 30-s maximal cycling bouts	Serum at rest, pre- and 48 h post-training	LC-MS	Targeted (296 lipids)	1	22:5 monoacylglycerol, DG (14:0/16:0, 16:0/16:0, 16:0/18:0, 16:1/18:2, 18:0/18:0), TG (50:0, 52:2, 54:1, 54:2), PI (16:0/18:0, 18:0/18:0), ceramides (d18:1/16:0, d18:1/18:1), hexosylceramide (d18:1/14:0, d18:1/16:1, d18:1/18:2) NEFA (12:1, 14:0, 14:1, 16:2, 18:3, 18:4, 20:4, 20:5, 22:3, 22:5, 22:6), 18:1/18:3 DG, 20:0/20:4 PI, 20:0 lysophosphatidylcholine, 18:2 lysophosphatidylethanolamine, 18:2 cholesteryl ester
10	Dong et al. (2024)	10 physically active men	SIE: 4 30-s maximal cycling bouts	Plasma, pre- and post-exercise	LC-MS	Targeted (15 metabolites), untargeted	1 (targeted) and 2 (untargeted)	Coriose, methoxyacetate, acetylcarnitine, propionylcarnitine, 18:0 lysophosphatidylserine, hypoxanthine, xanthine 6-Methyl-caprylate, 2-(ethylsulfinylmethyl)phenyl methylcarbamate

DG, diacylglycerol; LC, liquid chromatography; MAV; maximal aerobic velocity, MS, mass spectrometry; NMR; nuclear magnetic resonance spectroscopy; PI, phosphatidylinositol; SIE, sprint interval exercise; SIT, sprint interval training; TG, triacylglycerol; VO_2_peak, peak oxygen consumption; , increase; , decrease

### HIIE/HIIT

Table 1 presents the main information drawn from the 11 selected studies on HIIE/HIIT, listed in ascending chronological order. Despite using the same exercise type, the studies had different aims, which were reflected in their experimental design. A first distinction can be made between the five studies that employed one HIIE session and the six that employed HIIT. These two subsets are analyzed separately in the following subsections.

#### HIIE

Four of the studies that examined HIIE used trained or physically active males who performed either cycling or running protocols. One used both male and female athletes who completed roller skiing bouts. The biological samples selected by the researchers were plasma/serum, urine, or capillary blood.

Using a 60-min cycling protocol, Peake et al. (2014) noticed post-exercise increases in plasma glucose, lactate, citrate, aconitate, succinate, malonate, 3-methyl-2-oxovalerate, 2-oxoisocaproate, 7 non-esterified fatty acids (NEFA), and some amino acids. After the 2-h recovery period only two NEFA remained elevated, while several amino acids decreased below baseline (pre-exercise).

Using two running protocols, inversely related in terms of bout duration and intensity, Zafeiridis et al. (2016) found similar post-exercise increases in plasma pyruvate, glucose, alanine, lactate, glycerol, citrate, and succinate.

Zhao et al. (2020) investigated the effect of a yo-yo running protocol to exhaustion on the urinary metabolome. Comparison of the 30-min post-exercise to the pre-exercise samples indicated increases in arginine, proline, glutamate, citrulline, N-acetylornithine, meso-diaminoheptanedioate, N-acetyl-diaminoheptanedioate, γ-glutamylputrescine, 2-oxo-4-hydroxy-5-aminovalerate, and 4-hydroxyglutamate semialdehyde. By contrast, androstenedione and estrone decreased. No differences were spotted between the pre- and 18-h post-exercise samples.

Cruz et al. (2022), using a 20-min cycling protocol and analyzing the serum metabolome, found increased 2-hydroxyvalerate, 3-methyl-2-oxovalerate, N-acetylserotonin, oxypurinol, and valerate, as well as decreased acetone, carnitine, lysine, and thymine 10 min post-exercise compared with pre-exercise. Additionally, they found increased hypoxanthine and oxypurinol, as well as decreased 3-methylxanthine, acetone, lysine, and succinylacetone 60 min post-exercise compared with pre-exercise.

Karlsson et al. (2024), using a 45- to 60-min roller-ski treadmill test, found increases in blood lactate, pyruvate, uridine, malate, succinate, uracil, glycerate, xanthosine, hypoxanthine, ribose 5-phosphate, xanthine, fructose 6-phosphate, cyclic AMP, 9 acylcarnitines, inosine, and glucose 6-phosphate. They also found decreased 3-phosphoglycerate, phosphoenolpyruvate, AMP, uridine 5’-monophosphate, and deoxycholate.

In addition to the HIIE studies described above, the study by Kuehnbaum et al. (2015) examined acute as well as training effects, thus raising the number of studies on the acute effects of HIIE to six. Specifically, the authors performed a metabolomic analysis of plasma obtained from overweight or obese women before, as well as 20 and 40 min after a HIIE cycling protocol in the first and 17th training sessions. In both sessions, the post-exercise samples (at both 20 and 40 min) had higher acetylcarnitine and hypoxanthine concentrations, compared with baseline.

#### HIIT

Of the six studies that examined HIIT, five used sedentary men and women, while one used trained men. The biological samples analysed were plasma/serum, urine, or muscle tissue.

Kuehnbaum et al. (2014) applied a 6-week cycling program and, by comparing post- vs. pre-training plasma samples obtained in the resting state, found increases in ornithine, proline betaine, and trimethyllysine, as well as decreases in cysteinylglycine-cysteine disulfide, glutathione-cysteine disulfide, and cystine. In a study employing the same intervention but with blood sampling both before and after the first and penultimate training sessions (Kuehnbaum et al., 2015a), the authors found that training increased carnitine and, again, decreased glutathione-cysteine disulfide at rest, while decreasing hypoxanthine in the post-exercise samples.

Kistner et al. (2019) investigated the effect of a ten-day cycling program on the resting urinary metabolome of trained men. Compared with a non-training control group, they found a lower hypoxanthine concentration one day post-training and lower betaine, hypoxanthine, and isoleucine concentrations four days post-training.

Jurado-Fasoli et al. (2022) found that a 12-week program of running and weight-bearing exercises did not modify the plasma concentration of oxylipins or endocannabinoids at rest when compared with a non-training control group.

Moreover, Youssef et al. (2023), following a 12-week intervention on an elliptical device, found increases in 2-oxoglutarate, fumarate, ketoisovalerate, stearoylcarnitine, and margarate, as well as decreases in 3-methylhistidine, aspartate, acetate, glycerate, 2-hydroxybutyrate, 2-oxovalerate, inosine, acetylcarnitine, a ceramide, two diacylglycerols, isobutyrate, linoleate, and two acyl-alkyl-phosphatidylcholines in serum at rest.

Lastly, Pataky et al. (2024), after implementing a 12-week cycling intervention, found increases in muscle citrate, 2-oxoglutarate, 2-hydroxyglutarate, succinate, fumarate, malate, glutamate, phenylalanine, γ-aminobutyrate, aspartate, asparagine, methionine, serine, leucine, isoleucine, valine, 6 acylcarnitines, sphingosine, and 4 ceramides. The authors also found decreases in lysine, citrulline, sarcosine, 1-methylhistidine, carnitine, propionylcarnitine, and isovalerylcarnitine after the intervention.

### SIE/SIT

Table 2 summarizes the main information drawn from the nine selected studies on SIE/SIT, listed in ascending chronological order. Similarly to Sect. 3.1, five of the studies employed one SIE session and four employed SIT.

#### SIE

The five studies that examined SIE used physically active males who performed either cycling or running protocols. The biological samples varied between plasma and urine.

Pechlivanis et al. (2010) investigated changes in the urinary metabolome elicited by two exercise sessions differing in the duration of the rest interval between twin sprints (10 s vs. 1 min). In both sessions, they found that the metabolites altered by exercise were lactate, 2-hydroxyisovalerate, 2-oxoisocaproate, 3-hydroxyisobutyrate, 3-methyl-2-oxovalerate, 2-oxoisovalerate, 2-hydroxybutyrate, alanine, pyruvate, 2-oxoglutarate, inosine, fumarate, and hypoxanthine, all of which increased. A decrease after exercise was noticed in citrate, trimethylamine *N*-oxide, taurine, glycine, allantoin, phenylalanine, hippurate, tryptophan, and formate. Some metabolites differentiated the 2 exercise protocols by exhibiting higher alteration in the short-interval protocol (lactate, 2-hydroxybutyrate, 2-oxoisocaproate, 3-methyl-2-oxovalerate, 2-oxoisovalerate, alanine, pyruvate, citrate, 2-oxoglutarate, and fumarate) or in the long-interval protocol (glycine and hypoxanthine).

In a similar study, Pechlivanis et al. (2015) monitored the response of the urinary metabolome to three sprint runs at different times after the exercise. The results at 1-hour post-exercise showed increases in lactate, 2-hydroxyisovalerate, 2-hydroxybutyrate, 2-oxoisocaproate, 3-methyl-2-oxovalerate, 3-hydroxyisobutyrate, 2-oxoisovalerate, 3-hydroxybutyrate, 2-hydroxyisobutyrate, alanine, pyruvate, fumarate, acetate, hypoxanthine and inosine, accompanied by decreases in urate, valine, succinate, citrate, trimethylamine, trimethylamine *N*-oxide, tyrosine, formate, creatinine, glycine, 4-aminohippurate, and hippurate. The results at 1.5 h post-exercise showed increased lactate, acetate, hypoxanthine, inosine, and urate, accompanied by decreased valine, isoleucine, succinate, methylamine, trimethylamine N-oxide, tyrosine, formate, creatinine, glycine, 4-aminohippurate, and hippurate, all compared with baseline. Finaly, in the last sampling, at 2 h postexercise, hypoxanthine and inosine remained elevated, whereas creatinine, glycine, 4-aminohippurate, and hippurate remained below baseline.

Danaher et al. (2016) examined the metabolic perturbations in plasma following 2 workload-matched supramaximal cycling protocols, which thus qualify as SIE (although the authors characterize them as “high intensity exercise”). The first protocol (at 150% VO_2_peak) elicited increases in lactate, malate, and citrate during exercise and in the same metabolites plus alanine immediately post-exercise. At 1-hour post-exercise, malate, citrate, and xylose were above baseline, whereas sorbose and cholesterol were below baseline. The second protocol (at 300% VO_2_peak) elevated lactate, malate, and cholesterol during exercise while suppressing citrate. Immediately post-exercise, lactate, malate, sorbose, fructose, and cholesterol were higher than baseline, whereas asparagine, 2 NEFA, erythronate, xylitol, and xylose were lower. Finally, at 1-hour post-exercise, lactate and alanine were still above, whereas glutamate, asparagine, lysine, three NEFA, erythronate, xylitol, and xylose were below baseline.

Zhou et al. (2022) tested the effects of sodium bicarbonate supplementation on sprinting performance. Unfortunately, the authors presented no data regarding the effects of exercise on the urinary metabolome (instead, they compared only sodium bicarbonate to placebo).

Dong et al. (2024) examined the impact of four 30-s maximal cycling bouts on the plasma metabolome. The results showed increases in coriose, methoxyacetate, acetylcarnitine, 18:0 lysophosphatidylserine, propionylcarnitine, hypoxanthine, and xanthine, as well as decreases in 6-methyl-caprylate and 2-(ethylsulfinylmethyl)phenyl methylcarbamate.

In addition to the SIE studies described above, the study by Pechlivanis et al. (2013) examined acute as well as training effects, thus raising the number of studies on the acute effects of SIE to six. Specifically, the authors performed a metabolomic analysis of serum obtained from moderately trained men before and 30 min after a sprint running protocol in the first and last weeks of training. In both sessions, the post-exercise samples had higher lactate, pyruvate, and alanine concentrations, as well as lower leucine, valine, isoleucine, arginine/lysine, and glycoprotein acetyl concentrations, compared with the baseline.

#### SIT

The four studies that examined SIT used males at varying training states, who performed either cycling or running protocols. The biological sample was serum in three studies, while both serum and muscle tissue were analysed in one study.

Pechlivanis et al. (2013) monitored the response of the serum metabolome to sprint running training. The authors summarize the effects of training as increases in methylguanidine, citrate, glucose, valine, taurine, trimethylamine N-oxide, choline-containing compounds, histidines (including histidine, 1-methylhistidine, and 3-methylhistidine), and acetoacetate/acetone, as well as decreases in lactate, pyruvate, glycoprotein acetyls, and lipids (in general).

The three remaining studies used training with all-out Wingate cycle sprints. Aird et al.(2021) obtained blood and muscle tissue samples pre- and post-exercise, as well as pre- and post-training, but did not analyse data regarding the effects of exercise or training (instead, they presented data only on the impact of protein supplementation). Darragh et al.(2023) found decreased concentrations of 11 NEFA in the post-training serum samples at rest, compared with pre-training. Lastly, Wang et al. (2023), in their lipidomic analysis found increased concentrations of a monoacylglycerol, five diacylglycerols, four triacylglycerols, two phosphatidylinositols, two ceramides, and three hexosylceramides in the post-training serum samples at rest, compared with pre-training. By contrast, the concentrations of 11 NEFA, one diacylglycerol, one phosphatidylinositol, one lysophosphatidylcholine, one lysophosphatidylethanolamine, and one cholesteryl ester decreased after training.

### Pathway analysis

**Fig. 2:**
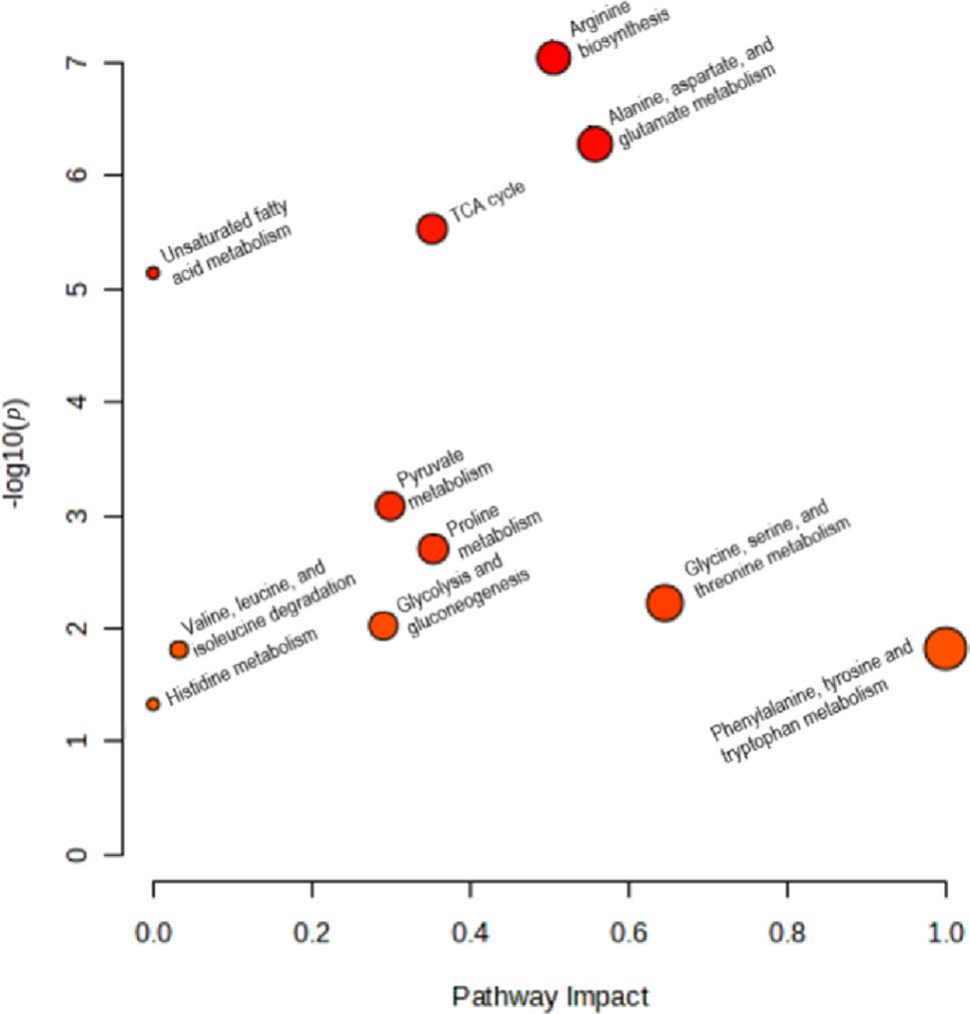
Pathway analysis, showing the main metabolic pathways affected by HIIE/HIIT or SIE/SIT according to the studies presented here. Each node represents a metabolic pathway. Pathway impact (x axis) is determined through pathway topology analysis, reflecting how central the affected metabolites are within the network. Node sizes are proportional to pathway impact. The y axis indicates the statistical significance of pathway alterations in the form of -log10(*p*) values. Values above 1.3 correspond to *p* < 0.05

Pathway analysis was performed with the aim of putting the findings on HIIE/HIIT and SIE/SIT metabolic perturbations in the context of biochemical pathways. This approach first uses over-representation analysis to determine whether a pathway contains more significant metabolites than expected by chance. These results are then combined with pathway topology analysis, which assesses the relative importance of the affected metabolites within the pathway, taking into account their centrality in the network (Xia. et al., 2011). Figure 2 presents an overview of the results based on the identified metabolites as significant markers of response to HIIE/HIIT or SIE/SIT. More detailed information about pathway analysis results can be found in Table S1. It can be seen that several pathways were modified, mostly related to carbohydrate, lipid, and amino acid metabolism, as well as the tricarboxylic acid (TCA) cycle.

## Discussion

The present work focuses on the metabolomic changes observed in response to HIIE/HIIT and SIE/SIT protocols. To interpret these findings in context, it is important to recognize broader methodological factors that can influence study outcomes. Variations in study designs may influence the overall conclusions of this review: Differences in the biological matrices analyzed (i.e., whole blood, serum, plasma, muscle, or urine) can affect the results, as each matrix produces distinct metabolomic signals. The timing of sample collection relative to exercise is also critical: sampling immediately after an exercise bout versus after a recovery period can reveal different metabolomic responses. Likewise, the intensity and duration of both exercise and recovery intervals can substantially alter the measured outcomes. Additionally, different dietary manipulations may produce distinct metabolic outcomes. Moreover, metabolomics results are inherently constrained by the portion of the metabolome detectable with a given analytical method (Khoramipour et al., 2022). Consequently, the findings summarized here should be regarded as complementary, with each study providing unique insights shaped by its experimental design and methodology.

Metabolite annotation is a major intrinsic limitation of metabolomic studies that can hinder efforts to synthesize findings across multiple investigations. In our analysis, inaccuracies in the original studies could directly influence pathway analysis results, as noted in recent reviews (Ong et al., 2023). This is why minimum reporting standards regarding metabolite annotation have been proposed to enhance the quality of metabolomic studies (Sumner et al., 2007). Notably, approximately half of the studies included in our work employed targeted approaches, using methods developed with analytical standards. Among the untargeted studies, five utilized in-house libraries for annotation and reported level 1 identifications, complemented by level 2 annotations derived from comparisons with public libraries. The large proportion of metabolites confirmed through analytical standards strengthens the confidence in our findings, while comparisons of results across different research groups provide further support for their reliability.

The previous consideration highlights the potential bias introduced by the current reliance on existing metabolite libraries and databases. Because only a fraction of the metabolome can be present in such resources, studies are likely to repeatedly identify the same compounds, potentially overlooking biologically significant features that have not been identified. This bias is further reinforced when targeted platforms are used, as they limit detection to predefined panels of metabolites. Consequently, signals without confident annotation are often underreported or disregarded. Although this limitation is not unique to our analysis, it highlights a critical challenge for the field: the need to develop strategies that capture and interpret the vast, currently uncharacterized portion of the metabolome.

This review examines the metabolic alterations that arise in response to HIIE/HIIT and SIE/SIT exercise protocols trough pathway analysis. Results were primarily considered significant at a raw *p* < 0.05. To facilitate interpretation, these pathways were grouped according to related biological functions. Because metabolic pathways are highly interconnected, some pathways with Holm-adjusted or false discovery rate *p* values above 0.05 were nonetheless included in the discussion when their raw *p* values met this threshold. The complete results of the pathway analysis can be found in Supplementary Table S1. Based on this analysis and the findings of the 20 studies reviewed, the metabolomic changes are discussed in 5 groups: carbohydrate metabolism, lipid metabolism, amino acid metabolism, TCA cycle, and purine degradation.

### Carbohydrate metabolism

**Fig. 3:**
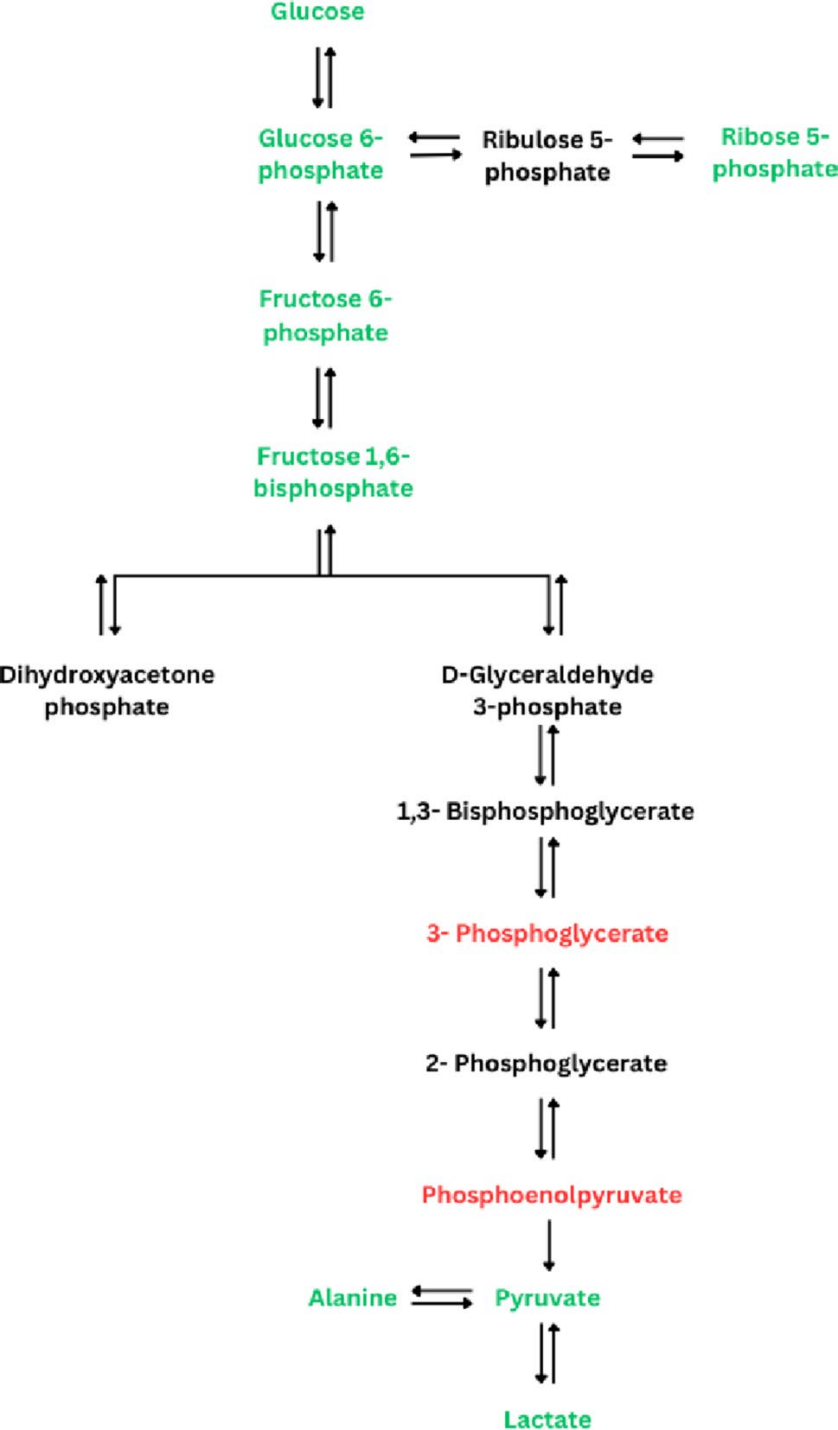
Effects of HIIE/HIIT and SIE/SIT on carbohydrate metabolism. Green represents an increase, red represents a decrease from baseline, and black represents metabolites which were not affected by exercise in any study. See Tables 1 and 2 for details

As can be expected from the carbohydrates’ pivotal role in exercise metabolism, the glycolytic/gluconeogenic metabolites are affected by HIIE/HIIT and SIE/SIT (Fig. 3). Several studies examined in this review reported increased glucose (Peake et al., 2014; Zafeiridis et al., 2016), pyruvate (Pechlivanis et al., 2010a, 2013a, 2015b; Zafeiridis et al., 2016) and lactate concentrations (Pechlivanis et al., 2010a, 2013a, 2015b; Zafeiridis et al., 2016) after HIIE or SIE, in accordance with the findings of numerous studies on high-intensity exercise (Mougios, 2019). The increase in glucose was attributed to enhanced glycogen breakdown in the liver during exercise (Zafeiridis et al., 2016), while the increase in pyruvate and lactate was interpreted as a clear sign of high glycolytic flux in muscle (Karlsson et al., 2024; Peake et al., 2014). However, Pechlivanis et al. (2013) also noticed decreased pyruvate and lactate in resting and post-exercise serum samples after SIT. The authors argue that training might have facilitated the removal of these metabolites from the blood. The observed increase in alanine concentration (Danaher et al., 2016; Peake et al., 2014; Pechlivanis et al., 2010a, 2013a, 2015b; Zafeiridis et al., 2016) can be attributed to the increase in pyruvate (with which alanine is connected through transamination).

The authors of one study (Karlsson et al., 2024) detected changes in 4 glycolytic/gluconeogenic intermediates (glucose 6-phosphate, fructose 6-phosphate, 3-phosphoglycerate, and phosphoenolpyruvate) and a related sugar phosphate (ribose 5-phosphate) without commenting on possible mechanisms. We believe that the rise in the “early” glycolytic intermediates (glucose-6-phosphate and fructose 6-phosphate) and ribose 5-phosphate can be explained by the rise in glucose and the well-known upregulation of glycogenolysis (whose product, glucose 1-phosphate, isomerizes to glucose 6-phosphate) by exercise. The contrasting drop in the “late” glycolytic intermediates, that is, 3-phosphoglycerate (which, to our knowledge, has not been reported with any exercise protocol before), and phosphoenolpyruvate (which has been reported in human erythrocytes (Ohno, 1978), human serum (Ohno, 1983), and rat muscle (Dohm et al., 1986), all after prolonged endurance exercise) may be due to exercise-induced activation of glycolytic enzymes, notably pyruvate kinase (Mougios, 2019).

### Lipid metabolism

**Fig. 4:**
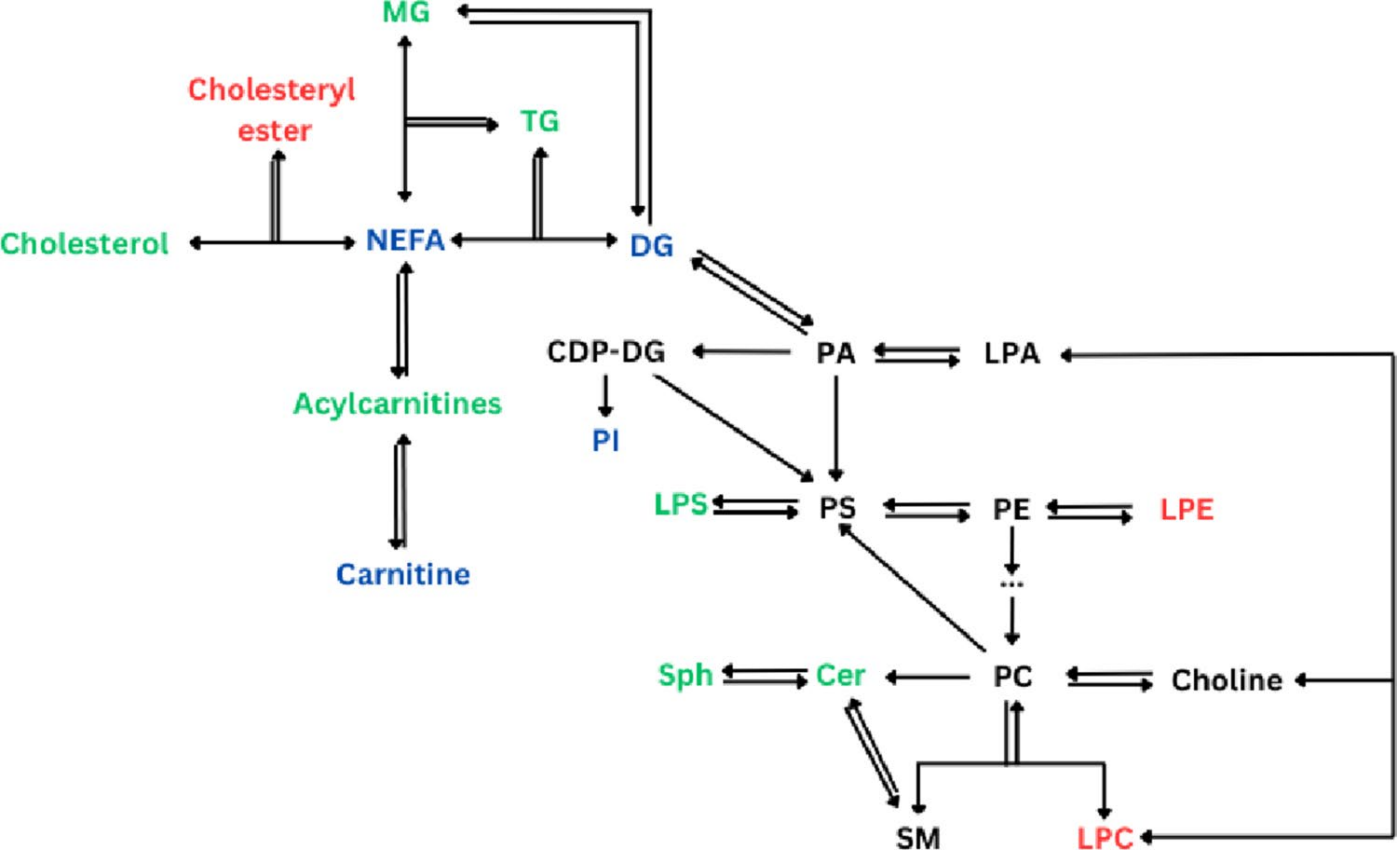
Effects of HIIE/HIIT and SIE/SIT on lipid metabolism. Green represents an increase (in all or most studies), red represents a decrease, black represents metabolites which were not affected by exercise in any study, and blue represents mixed results (increase in some studies and decrease in others). See Tables 1 and 2 for details. CDP-DG, cytidine diphosphate diacylglycerol; Cer, ceramides; DG, diacylglycerol; LPA, lysophosphatidic acid; LPC, lysophosphatidylcholine; LPE, lysophosphatidylethanolamine; LPS, lysophosphatidylserine; MG, Monoacylglycerol; NEFA, non-esterified fatty acids; PA, phosphatidic acid; PC, phosphatidylcholine; PE, phosphatidylethanolamine; PI, phosphatidylinositol; PS, Phosphatidylserine; SM, sphingomyelin; Sph, sphingosine; TG, triacylglycerol

The use of fatty acids as an important energy source for muscle during many forms of exercise and the mobilization of triacylglycerols as the main fatty acid suppliers through lipolysis result in remarkable perturbations in lipid homeostasis, both acutely and chronically (Bosma, 2014; Muscella et al., 2020). Pathways and metabolites related to lipid metabolism that were found to be affected by HIIE/HIIT and SIE/SIT in the present review are summarized in Fig. 4.

As can be seen in Fig. 4; Tables 1 and 2, the plasma/serum concentrations of NEFA exhibited mixed responses to HIIE/HIIT and SIE/SIT. It should be noted that most of the individual NEFA determined in the relevant studies (Danaher et al., 2016; Dong et al., 2024; Peake et al., 2014; Wang et al., 2023; Youssef et al., 2023; Zhao et al., 2020) did not change significantly, reflecting a general balance between supply to the circulation from adipose tissue and uptake by the exercising muscles. Of the NEFA that changed significantly, most decreased. Regarding the acute effects, there was a distinction between HIIE, which caused increases (Peake et al., 2014), and SIE, which caused decreases (Danaher et al., 2016; Dong et al., 2024). This is probably due to the different intensity and, particularly, duration of the exercise protocols (40 in HIIE (Peake et al., 2014) vs. 5–2 min in SIE (Danaher et al., 2016; Dong et al., 2024). Indeed, it is known that moderate or hard exercise causes an initial (up to 10 min) drop, followed by an increase in the circulating NEFA concentration (Romijn et al., 1993). Regarding the chronic effects, the opposing effects of HIIT in just 2 NEFA (Youssef et al., 2023) suggest no considerable effect on NEFA as a whole. On the other hand, the downregulation of 11 NEFA at rest in each of the 2 studies with short-term (6- or 3-week) SIT (Darragh et al., 2023; Wang et al., 2023) points to potential adaptations such as reduced release to the blood and/or increased tissue uptake. The possibility of reduced NEFA release to the blood in response to SIT is corroborated by the increase in several serum triacyclglycerols, diacylglycerols, and a monoacylglycerol, concomitant with the decrease in NEFA in one of these two studies (Wang et al., 2023), since acylglycerols are efficient stores of fatty acids.

Another class of compounds closely related to fatty acid metabolism is acylcarnitines. These are essential for transferring long-chain fatty acids across the inner mitochondrial membrane for subsequent β oxidation. The acute increase in not only long-chain but also medium- and short-chain acylcarnitines found unanimously after both HIIE (Karlsson et al., 2024; Kuehnbaum et al., 2015a) and SIE (Dong et al., 2024) is apparently due to the increased availability of NEFA as substrates for acylcarnitine synthesis. The acute decrease in (free) carnitine after HIIE (Cruz et al., 2022) agrees with this hypothesis. As for the effects of training, these seem to depend on chain length, since both studies with HIIT have found decreases in short-chain acylcarnitines and increases in long-chain ones (Pataky et al., 2024; Youssef et al., 2023).

Ceramides, a group of lipids that are known as signaling molecules involved in inflammation mechanisms, with several implications such as cardiovascular disease and autoimmune disorders (Chaurasia & Summers, 2025; Ding et al., 2024) mostly increased with HIIT or SIT (9 species increased (Pataky et al., 2024; Wang et al., 2023), as opposed to 1 species decreasing (Youssef et al., 2023). The increase in sphingosine (Pataky et al., 2024), a structurally related metabolite, may reflect the increase in ceramides. The physiological significance of these findings (given the well-documented beneficial effects of exercise training) is unclear. The increases in ceramides have been attributed to exercise-induced inflammation (Wang et al., 2023), although this might not be expected to persist for 2 or 3 days after the last training session (when sampling was performed). It would be interesting to determine whether the increases seen reach pathological levels.

Lastly, the following lipids shown in Fig. 4 were reported to change in only 1 study each: phosphatidylinositol, lysophosphatidylcholine, lysophosphatidylethanolamine, and cholesteryl ester (Wang et al., 2023); cholesterol (Danaher et al., 2016); and lysophosphatidylserine (Dong et al., 2024). This, combined with the fact that several connecting lipids in their pathways have not been determined, makes it precarious to draw conclusions regarding the effects of HIIE/HIIT and SIE/SIT on them.

### Αmino acid metabolism

**Fig. 5:**
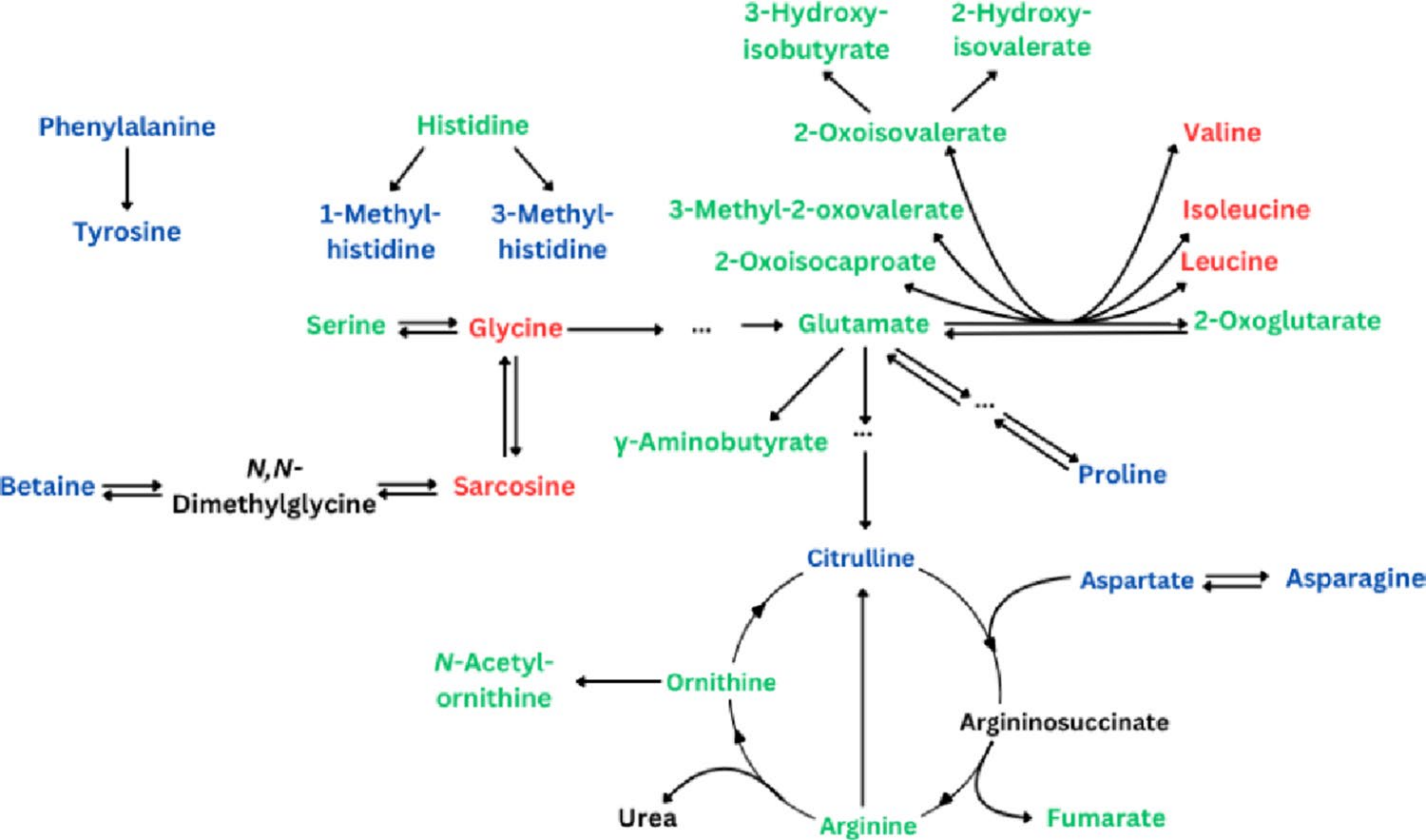
Effects of HIIE/HIIT and SIE/SIT on amino acid metabolism. Green represents an increase (in all or most studies), red represents a decrease (in all or most studies), black represents metabolites which were not affected by exercise in any study, and blue represents mixed results (Increase in some studies and decrease in others). See Tables 1 and 2 for details

The concentrations of amino acids and related metabolites in bodily fluids are frequently found to be affected by exercise, and this was also the case with many of the studies reviewed here. The pathways involving these compounds and how HIIE/HIIT and SIE/SIT modified their concentrations can be seen in Fig. 5.

The most consistent effects of HIIE/HIIT and SIE/SIT on amino acid metabolism involved the branched-chain amino acids (BCAA), leucine, isoleucine, and valine (upper right corner of Fig. 5). Their concentrations decreased (Peake et al., 2014; Pechlivanis et al., 2013a, 2015b) concomitant with increased concentrations of the products of their transamination and further reactions (Cruz et al., 2022; Peake et al., 2014; Pechlivanis et al., 2010a, 2013a, 2015b; Youssef et al., 2023). Such findings indicate an increase in BCAA degradation. The finding of increased BCAA concentrations (Pataky et al., 2024) refers to a training effect, as opposed to acute decreases (Peake et al., 2014; Pechlivanis et al., 2013a, 2015b). Thus, these results are not contradictory. A study that employed combined endurance training and HIIT detected an increase only in plasma isoleucine among the BCAA (Lee et al., 2021). Interestingly, the authors found a concomitant improvement in insulin sensitivity, which is against the widely held notion of a positive link between the plasma BCAA concentration and insulin resistance.

Two metabolites participating in all transamination reactions, 2-oxoglutarate and glutamate, increased with HIIE/HIIT and SIE/SIT (Pataky et al., 2024; Peake et al., 2014; Pechlivanis et al., 2010a; Youssef et al., 2023; Zhao et al., 2020), probably reflecting the increased flux through the TCA cycle, of which 2-oxoglutarate is an intermediate (see next subsection). Other metabolites linked to glutamate in Fig. 5 have shown mixed responses and/or have been reported in just 1 or 2 studies. This does not lay a safe ground to discuss their responses to HIIE/HIIT and SIE/SIT. The same is true of the phenylalanine-tyrosine pair (mixed responses in 2 studies per metabolite) and the paths connecting histidine (reported in one study (Pechlivanis et al., 2013a) with 1- and 3-methylhistidine (mixed responses in two studies per metabolite).

Few of the studies reviewed in this work have reported changes in metabolites connected with the urea cycle (bottom of Fig. 5), except for fumarate, which seems to exhibit a robust increase with HIIT (Pataky et al., 2024; Youssef et al., 2023) and SIE (Pechlivanis et al., 2010a, 2015b). The other metabolites showed increases or mixed results. This suggests that, if anything, the urea cycle is upregulated, consistent with its role of incorporating potentially hazardous ammonia into innocuous urea and with the rise in plasma ammonia after strenuous exercise (Ring, 1999; Brouns et al., 1990; Morris, 2002).

The findings discussed in this subsection highlight the diversity of responses of amino acids and their metabolites to HIIE/HIIT and SIE/SIT. Perturbations in relevant pathways may be the basis for important effects on exercise metabolism. In addition, one should not forget how interconnected these pathways are with other pathways, such as glycolysis (subsection 4.1, where alanine was discussed due to its interconversion with pyruvate) and the TCA cycle (next subsection, where 2-oxoglutarate and fumarate are also included).

### TCA cycle

**Fig. 6:**
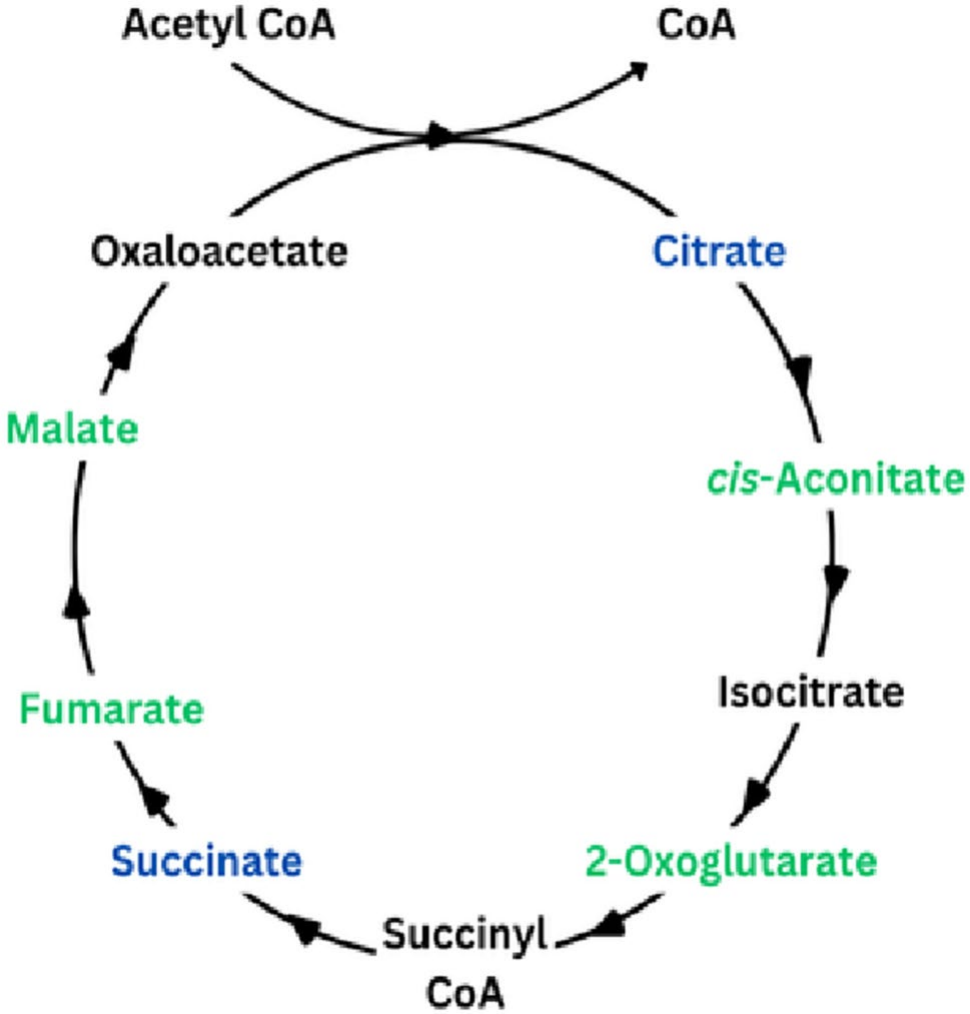
Effects of HIIE/HIIT and SIE/SIT on metabolites of the TCA cycle. Green represents an increase, black represents metabolites which were not affected by exercise in any study, and blue represents mixed results (Increase in some studies and decrease in others). See Tables 1 and 2 for details

The TCA cycle, also known as the Krebs cycle or citric acid cycle, plays a central role in energy production and is therefore expected to be greatly affected by exercise. An overview of the TCA cycle, with an indication of the metabolites found to be affected by exercise in this review, is shown in Fig. 6.

All TCA cycle metabolites measured increased with HIIE/HIIT and SIE/SIT in all or most cases (Danaher et al., 2016; Karlsson et al., 2024; Pataky et al., 2024; Peake et al., 2014; Pechlivanis et al., 2010a, 2013a, 2015b; Youssef et al., 2023; Zafeiridis et al., 2016; Zhao et al., 2020). Such changes are justified by the acceleration of this pathway in muscle during exercise due to a multitude of factors, including increased entry of acetyl CoA (resulting from augmented glycolysis and β oxidation) and activation of key TCA cycle enzymes (Mougios, 2019), all increasing ATP production. In a minority of cases, all associated with SIE, citrate (Danaher et al., 2016; Pechlivanis et al., 2010a, 2015b) and succinate (Pechlivanis et al., 2015b) decreased, suggesting that changes in these metabolites may not be as clear as in other TCA cycle intermediates.

### Purine degradation

**Fig. 7:**
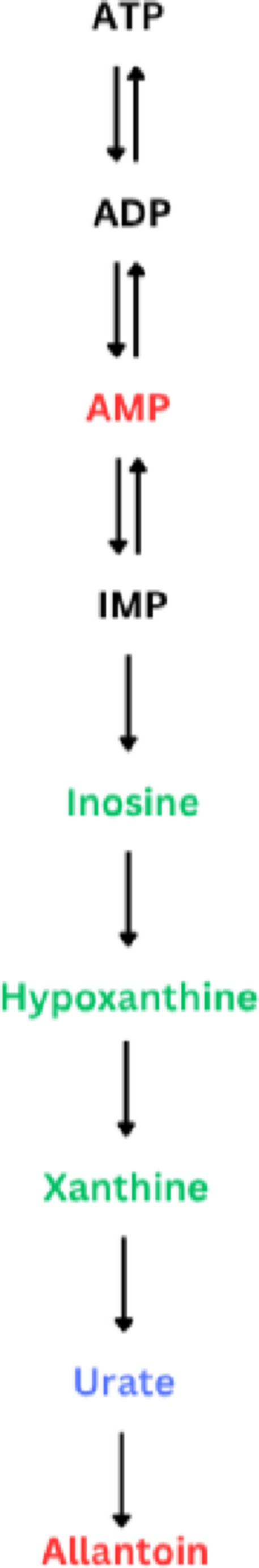
Effects of HIIE/HIIT and SIE/SIT on metabolites of purine degradation. Green represents an increase (in most or all studies), red represents a decrease, black represents metabolites which were not affected by exercise in any study and blue represents mixed results (increased in some studies while decreased in others). See Tables 1 and 2 for details. ADP, adenosine diphosphate; AMP, adenosine monophosphate; ATP, adenosine triphosphate; IMP, Inosine monophosphate

Although not present as a significantly affected metabolic pathway in Fig. 2, the pathway of purine degradation plays an important role in exercise metabolism, and several of its metabolites were affected by HIIE/HIIT or SIE/SIT (Cruz et al., 2022; Dong et al., 2024; Karlsson et al., 2024; Kistner et al., 2019; Kuehnbaum et al., 2015a; Pechlivanis et al., 2010a, 2015b; Youssef et al., 2023). An overview of the pathway and the affected metabolites is shown in Fig. 7.

The upregulation of purine degradation is closely linked to increased ATP hydrolysis in muscle during hard and maximal exercise (Zhao et al., 2000). Of this pathway’s metabolites, inosine, hypoxanthine, and xanthine were repeatedly and consistently found to increase after HIIE or SIE (Cruz et al., 2022; Dong et al., 2024; Karlsson et al., 2024; Kuehnbaum et al., 2015a; Pechlivanis et al., 2010a, 2015b), testifying to massive purine degradation. Of note, 3 studies found decreases in the resting (Kistner et al., 2019; Youssef et al., 2023) or postexercise (Kuehnbaum et al., 2015a) concentrations of inosine or hypoxanthine after HIIT, in accordance with the finding of attenuated muscle ATP degradation after sprint training (Stathis et al., 1994), which the authors attribute to improved balance between ATP hydrolysis and resynthesis. AMP (Karlsson et al., 2024) urate (Pechlivanis et al., 2015b), and allantoin (Pechlivanis et al., 2010a), were reported to change with HIIE or SIE in only one study each. Remarkably, urate presented a biphasic response, that is, decrease and then increase in urine after SIE, suggesting the use of plasma urate in antioxidant defense against reactive oxygen species produced in the immediate post-exercise period (Pechlivanis et al., 2015b). Overall, the findings presented highlight a perturbation of purine metabolism with intense and maximal exercise.

### Comments on study design

Based on our appraisal of the presented studies, we would recommend the inclusion of a non-exercising control group in future studies to distinguish the effects of the acute or chronic exercise intervention from confounding variables, such as diurnal or seasonal variation, thus ensuring that observed changes in metabolites are truly attributable to exercise. Remarkably, of the 20 selected studies, only two HIIT studies (Jurado-Fasoli et al., 2022; Kistner et al., 2019) included a control group that refrained from training, helping to better isolate the effects of exercise on metabolites.

An additional methodological consideration is the control of diet. Nutritional intake can act as an important confounder in exercises metabolomics. Some of the studies applied strict dietary protocols, others relied on participants maintaining habitual intake. Because of that, interindividual and interindividual dietary habits could have affected the metabolite responses. The lack of dietary control was acknowledged as a limitation in many of the studies, emphasizing on the need for more nutritional standardization in future studies.

### Limitations

The following limitations were discerned in our systematic review.

1. Although the tables and text in the Results section separate HIIE, HIIT, SIE, and SIT, metabolic perturbations are examined collectively in the discussion above. This unification was imposed by the small number of studies (four to six) in each case, which, combined with the large number of metabolites, would render the extraction of conclusions on each exercise or training mode separately unreliable.
2. Only statistically significant metabolite changes were presented, even though non-significant changes of the same metabolites were presented in other studies.
3. Annotation is an intrinsic limitation in metabolomics studies. Although the majority of the studies included in this review presented targeted methodologies or level 1 annotations, metabolites annotated with level 2 confidence were also included in pathway analysis.
4. Being a systematic review, this work is inherently constrained by the limitations of the original studies, including the small number of participants in several of them.

## Conclusion

The current systematic review included 20 carefully selected studies on the acute and chronic effects of two types of exercise, high-intensity interval and sprint interval, on the human blood, urine, and muscle metabolome. The studies identified tens of metabolites that changed with HIIE/HIIT or SIE/SIT. Notably, changes were observed in carbohydrate metabolism (glycolysis and gluconeogenesis), lipid metabolism (NEFA, acylglycerols, acylcarnitines, etc.), amino acid metabolism (especially pathways involving BCAAs), the TCA cycle, and purine degradation. These findings show a strong impact of such exercise modalities on human metabolism and underscore substrate utilization for energy production as a recurring theme. Furthermore, they reveal multiple interconnections of metabolites through these pathways.

On the other hand, it was shown that a wide variety of metabolites are reported to be affected by HIIE/HIIT or SIE/SIT, with few inconsistencies, as several factors, such as training protocol, timing of sampling, biological samples taken, and analytical platforms, affect the obtained metabolic fingerprints. Thus, reproducibility of the obtained results by metabolomic studies—a great challenge to this research area—should also be considered. Taking these concerns and the limitations listed above into account, the relatively small number of studies that met our inclusion criteria does not allow for a convergence of opinion regarding many of the observed responses at present. Finally, other modulating or confounding factors, such as diet and use of supplements, should be better controlled in future studies.

## Supplementary Information

Below is the link to the electronic supplementary material.

Supplementary Material 1

## Data Availability

No datasets were generated or analysed during the current study.
