# Supplementary material for: Metabolomic signatures of high-intensity and sprint interval exercise/training in humans: a systematic review
Source: Metabolomics. 2026 Feb 9;22(2):25. doi: 10.1007/s11306-025-02385-2 (PMC12886373; doi:10.1007/s11306-025-02385-2)
Supplement: Supplementary file 1 — Supplementary Material 1 [file 11306_2025_2385_MOESM1_ESM.docx]

Table 3 – Pathway analysis results

| Pathway | Hits* | Expected hits** | Raw p | -log(p) | Holm- adjusted p | FDR | Impact |
| --- | --- | --- | --- | --- | --- | --- | --- |
| Arginine biosynthesis | 8/14 | 0.73 | 9.10E-08 | 7.04 | 7.28E-06 | 7.28E-06 | 0.51 |
| Alanine, aspartate and glutamate metabolism | 10/28 | 1.46 | 5.26E-07 | 6.28 | 4.10E-05 | 1.40E-05 | 0.56 |
| Citrate cycle (TCA cycle) | 8/20 | 1.04 | 2.95E-06 | 5.53 | 2.24E-04 | 4.72E-05 | 0.35 |
| Biosynthesis of unsaturated fatty acids | 10/36 | 1.88 | 7.22E-06 | 5.14 | 5.42E-04 | 9.63E-05 | 0.00 |
| Pyruvate metabolism | 6/23 | 1.20 | 8.28E-04 | 3.08 | 6.04E-02 | 8.28E-03 | 0.30 |
| Arginine and proline metabolism | 7/36 | 1.88 | 1.98E-03 | 2.70 | 1.42E-01 | 1.76E-02 | 0.35 |
| Glycine, serine and threonine metabolism | 6/33 | 1.72 | 5.96E-03 | 2.22 | 4.17E-01 | 4.34E-02 | 0.65 |
| Glycolysis or Gluconeogenesis | 5/26 | 1.36 | 9.44E-03 | 2.03 | 6.51E-01 | 5.81E-02 | 0.29 |
| Phenylalanine, tyrosine and tryptophan biosynthesis | 2/4 | 0.21 | 1.50E-02 | 1.82 | 1.00E+00 | 8.22E-02 | 1.00 |
| Valine, leucine and isoleucine degradation | 6/40 | 2.09 | 1.54E-02 | 1.81 | 1.00E+00 | 8.22E-02 | 0.03 |
| Histidine metabolism | 3/16 | 0.83 | 4.68E-02 | 1.33 | 1.00E+00 | 2.20E-01 | 0.00 |

  FDR, false discovery rate

*Number of metabolites present in our analysis out of the total number of metabolites present in the pathway.

**The expected number of “hits” (significant metabolites) that would fall into a given pathway just by chance.
